# Mid-term results in revision hip arthroplasty with impaction bone grafted cup reconstruction for acetabular defects

**DOI:** 10.1038/s41598-022-17526-z

**Published:** 2022-08-03

**Authors:** Sebastian Rohe, Nicoletta Dörr, Sabrina Böhle, Georg Matziolis, Steffen Brodt, Eric Röhner

**Affiliations:** 1 Orthopaedic Department of the Waldkliniken Eisenberg, Orthopaedic Professorship of the University Hospital Jena, Campus Waldkliniken Eisenberg, Klosterlausnitzer Straße 81, 07607 Eisenberg, Germany; 2 Orthopaedic Department of the Heinrich-Braun-Hospital Zwickau, 08060 Zwickau, Germany

**Keywords:** Medical research, Outcomes research, Trauma, Musculoskeletal abnormalities

## Abstract

Acetabular defects are a challenging condition for surgeons in revision THA. A crucial aim is an anatomical restoration of the centre of rotation (COR) through grafts. The aim of this study was to determine the cup survival after biological restoration of acetabular defects in THA and the effect of Paprosky classification, age, BMI, and number of previous operations on cup survival. Retrospectively patients with a cup exchange and an impaction of cortico-cancellous or bulk grafts between 2009 and 2012 were included with a follow up with a minimum of 5 year. Implant failure was defined as radiographic loosening or explantation of the cup. The acetabular defect situation was classified to Paprosky. 82 patients (58 female 70.7%) were included. 26 patients were not available to contact. 56 patients (40 female 71.4%) remained for survival analysis with mean age of 75.6 ± 8 years. Survival of the cup after 5 years was 90% and after 7.8 years 88%. There was no difference in survival concerning defect classification, type of implant or graft, age, BMI, and number of previous operations. Patients on the follow up reached an HHS of 67.4 ± 19, a WOMAC Score of 33.4 ± 25.4 points and an unsatisfactory result in the SF-36. Impaction bone grafting of acetabular defects is a good option with satisfactory biomechanical results and survival for small defects. Predictive factors for cup survival could not be clarified in our study. So, the correct indication, knowing the limits of the methods and the correct choice of implant allow a defect-oriented approach and are decisive for the success of the operation.

## Introduction

Total Hip Arthroplasty (THA) is a common operation with increasing case numbers of primary and revision procedures over the last decades^1–3^. A severe complication after THA requiring revision is loosening of the cup, sometimes associated with a bony defect of the acetabulum and migration of the cup. A crucial aim of primary THA and revision surgery is an anatomical restoration of the centre of rotation (COR)^4^. Primary and secondary defects of the acetabulum can lead to a non-anatomical positioning of the cup with a potential higher rate of failure, while an anatomical biomechanical position of the cup increases longevity and decreases stress^4,5^. Slooff et al. described in the 1980s a technique of impaction bone grafting (IBG) for restoring the anatomical COR with bone grafts for a biological reconstruction with good short- and long-term survival^6–8^.

While using a biological graft for restoring the COR and establishing a new bony bed for the cup is advantageous, resorption, cup migration and a lesser primary stability in some cases are serious limitations of this technique. So metallic support like the Trabecular-Metal-Technology (Fa. Zimmer) was developed to reach a better primary stability with the disadvantage of implanting another foreign body, which can lead to an even greater bone defect in potential following revisions^9–11^.

Also, the type of biological grafts seems to play a significant role. Allogenic cortico-cancellous chips showed an incorporation through endochondral ossification and thus biological closure of the defect^12,13^. A primary mechanical stability is achieved by impacting and using cortico-cancellous chips. Studies using impacted bone chips and cementless cups for the reconstruction of moderate defects show satisfactory results with survival rates of over 90% after more than 10 years^14,15^. There is consensus that hemispherical press-fit cups should only be used if the cup circumference is intact and that there should be at least 50% contact surface with the recipient's vital bone^16^. But there is a lack of knowledge about the influencing factors for cup survival to make general recommendations. Another application form of allogenic bone grafting are bulk or solid allografts. They are used for large cavitary and segmental defects up to pelvic discontinuity^17–19^. The bone incorporation in bulk allografts takes place only partially in the revascularized edge zone of the transplant. Inadequate angiogenesis and the risk of resorption of the allograft are often seen as a limitation of their long-term stability and their use is therefore generally controversial^20^.

The aim of this study was (1) to determine the cup survival after biological restoration of acetabular defects in THA after a minimum follow-up of 5 years, (2) to evaluate the effect of the defect classification of Paprosky on the cup survival and to analyse the influence of age, Body-Mass-Index (BMI) and number of previous operations on cup survival and (3) to compare the Short-Form-36 health questionnaire (SF-36), Harris-Hip-Score and the Western Ontario and McMaster Universities Osteoarthritis Index (WOMAC-Score) after a minimum follow up of 5 years.

## Patients and methods

**Figure 1:**
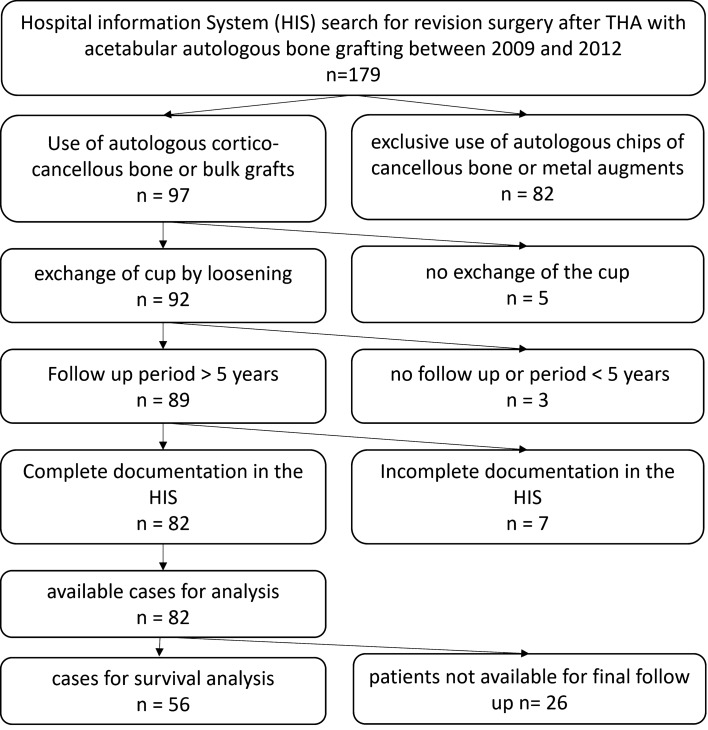
Flowchart of case inclusion and exclusion.

We retrospectively included all cases with previous THA and a planned cup exchange because of aseptic and septic loosening and an allogenic cortico-cancellous or bulk bone graft from our own bone bank between 2009 and 2012 (Fig. 1). Patients with an additional trabecular metal implant or cancellous bone grafts only were excluded. A minimal follow up of 5 years was determined. Patients were invited for a follow-up investigation with conventional a.p. and axial radiograph of the hip, clinical examination, SF-36-Score, HHS and WOMAC-Score. Patients refusing the invitation were interviewed by telephone. Therapy failure of cup survival was defined as radiographic loosening or explantation of the cup. The acetabular defect situation was determined on conventional a.p. radiographs of the hip to Paprosky classification^21^. Cup loosening was defined by a circumferent radiolucent line, cup migration and broken fixation screws^22–24^. The standardized Questionnaires were compared to a normal and age adjusted population.

The operation was done in lateral decubitus. The cup type and size were determined by a cup trial and subsequently a customized femoral head allograft from our own bone bank was used to minimize the defect in impaction graft technique. For small defects bone was prepared into small cortico-cancellous pieces of 5 to 8 mm. For greater defects (Paprosky > IIc) the bone was prepared into a bulk. Both were rinsed with a jet lavage with saline to wash out fatty remnants and rinsed with an antibiotic solution with gentamicin. The bone graft was impacted with cup trials and special impactors and subsequently the endoprosthetic treatment was completed. All patients received a perioperative antibiotic single shot and an additional shot if the operation time was longer than 2 h. In case of septic loosening the patients got a germ adapted antibiotic treatment over 12 weeks after operation. After operation the patient had a restrictive physiotherapeutic exercise program with a limited weight bearing of 20 kg or sole contact for 6 weeks. After 6 weeks an X-ray control was done and a gradually increase of weight bearing was started with additional 20 kg every 2 weeks for 6 weeks. After the next X-ray control the limitation of the weight bearing was ended and the crutched were trained off.

The study was approved by the independent ethics committee of the authors’ affiliated institution (IRB NO. 5283-09/2017) and informed consent was obtained from all participants. All procedures being performed were part of the routine care and in accordance with relevant guidelines and regulations.

### Statistical analyses

Statistical analysis was performed with the statistics program SPSS (Version 25, IBM). As an analytical approach a student’s t-test was performed for parametric tests of two samples and Log-Rank-test was performed for analysing the influence of confounders. The level of significance was *p* < 0.05. Investigating the survival of the implant a Kaplan–Meier-Analysis generally and regarding to the defect classification, age, BMI, and number of previous operations was performed.

### Ethics approval

Ethical approval was done by the local Ethics Committee of University Jena in view of the retrospective nature of the study. All the procedures being performed were part of the routine care (Reg.-Nr. 5283-09/2017).

## Results

We included retrospective 82 patients (24 male 29.3%, 58 female 70.7%) with an acetabular bone defect treated with an allogenic bone graft and a new cup. 26 patients were not available to contact for final follow up (Fig. 1). So, 56 patients (40 female 71.4%, 16 male 38.6%) remained for final survival analysis and 17 patients completed the questionnaires for analysing the patient’s outcome.

### Demographic data

**Figure 2:**
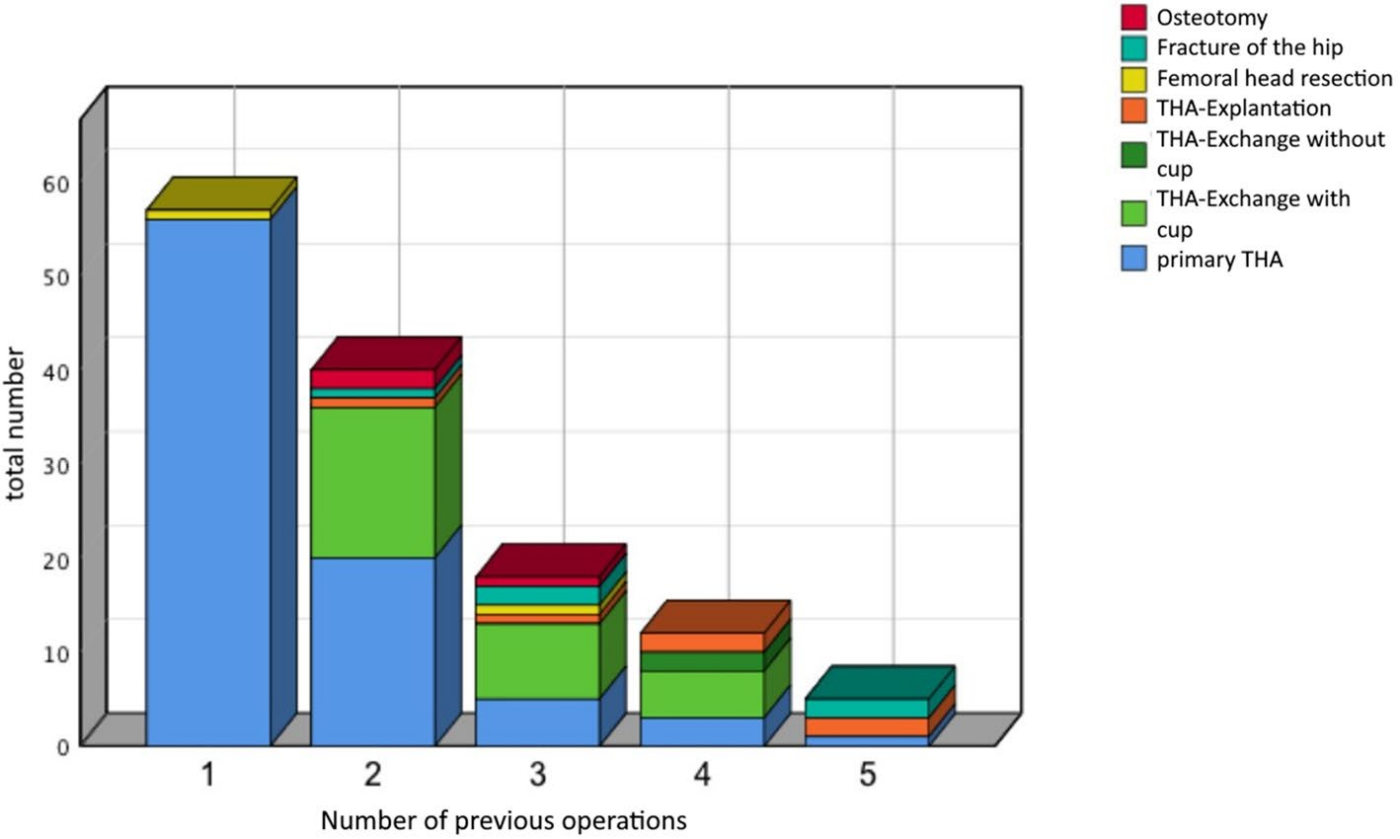
Previous operations before study index operation.

Mean age of the 82 patients at the index operation was 70.3 ± 9 years. Mean age of the 56 patients on follow up was 75.6 ± 8 years. Mean BMI at the index operation was 28 ± 4.8 kg/m^2^, while 30.5% had normal weight, 36.6% had overweight, 26.8% had obesity WHO I°, 3.7% WHO II° and 2.4% WHO III° (Table 1). All patients had a previous operation of the hip, 22.6% had two, 6.8% three, 3.4% four and 1.1% five previous operations (Fig. 2).

**Table 1:** Baseline characteristics.

	N	Complete	Male	Female
Age (years)	82	70.3 ± 9.0	69.1 ± 7.6	70.8 ± 9.5
Size (cm)	82	163.3 ± 9.7	171.8 ± 8.7	159.9 ± 7.8
Weight (kg)	82	74.8 ± 14.9	83.1 ± 15.5	71.3 ± 13.3
BMI (kg/m^2^)	82	28.0 ± 4.8	28.0 ± 4.4	27.9 ± 5.0
Normal weight	82	30.5%	29.2%	31.0%
Overweight	82	36.6%	33.3%	37.9%
Obesity I°	82	26.8%	33.3%	24.1%
Obesity II°	82	3.7%	4.2%	3.4%
Obesity III°	82	2.4%	0.0%	2.4%
**Secondary disease**				
Arterial hypertension	60	73.2%		
Coronary syndrome	9	11.0%		
Cardiac arrhythmias	8	9.8%		
Heart failure	5	6.1%		
Diabetes mellitus	17	20.7%		
Thyroid disease	14	17.1%		
Malignancy	7	8.5%		
COPD	5	6.1%		
Renal failure	3	3.7%		
Immunosuppression	3	3.7%		
Osteopenia or osteoporosis	19	23.2%		
Rheumatoid arthritis	7	8.5%

### Operation data

**Figure 3:**
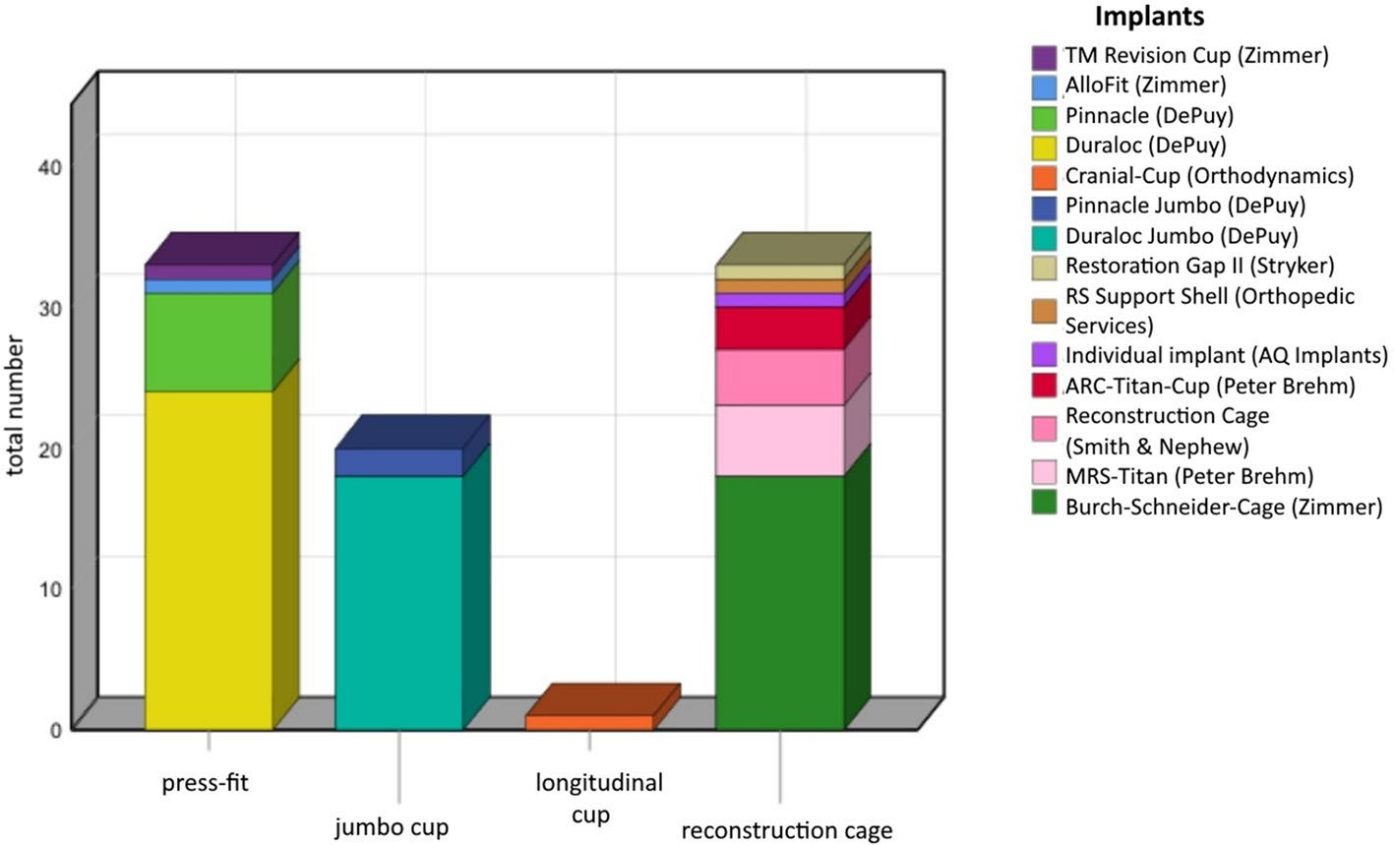
Categorized implants of implanted cups.

**Figure 4:**
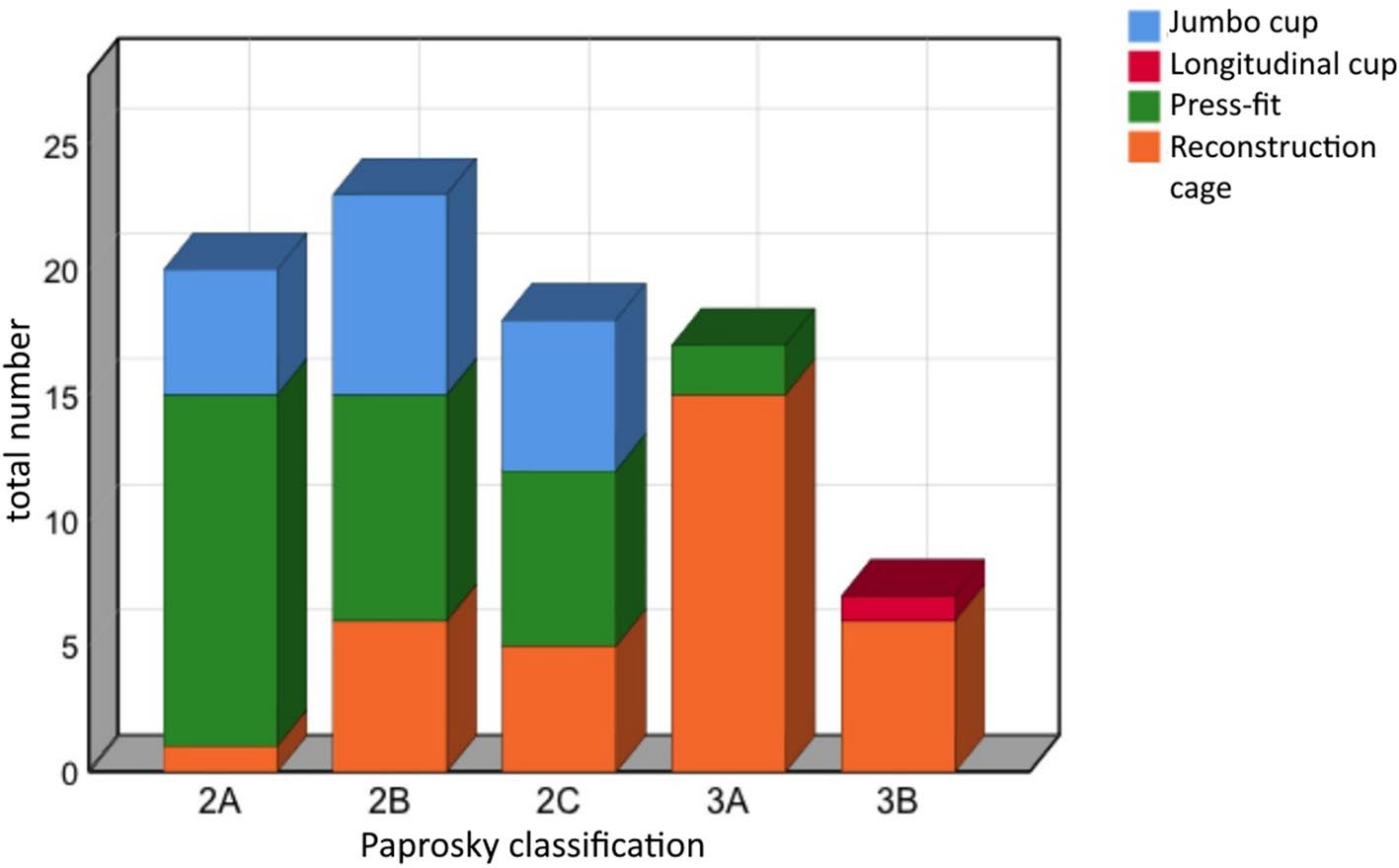
Revision strategy depending on Paprosky classification.

Indication for index revision surgery was aseptic loosening (81.6%), aseptic loosening in presence of osteolysis by wearing of the PE-inlay (8%), periprosthetic fractures (4.6%) and septic loosening (5.8%). Femoral head allografts from our bone bank were used in all cases. 25 patients (44.8%) had a solid structured bulk graft, 18 (32.2%) cortico-cancellous bone chips and 13 (23%) were treated with a combination of bulk and cortico-cancellous bone grafts. Implants used for endoprosthetic treatment were shown in Fig. 3 and depending on the Paprosky defect type in Fig. 4. Bone defects were classified after Paprosky at the preoperative a.p. radiographs with an occurrence of type 2a in 23.5%, type 2b 27.1%, type 2c 21.2%, 3a 20% and type 3b 8.2%, respectively.

### Implant survival

46 of 56 patients had no further operation (82%). 10 patients (18%) had complications after the cup exchange and bone grafting while some occurred in the same patient: 6 aseptic cup loosening (2 press fit cups, 2 jumbo cups, 2 revision cages), 4 periprosthetic infections, 2 recurrent dislocation, 1 periprosthetic fracture and 1 major limb amputation of the thigh on both sides.

**Figure 5:**
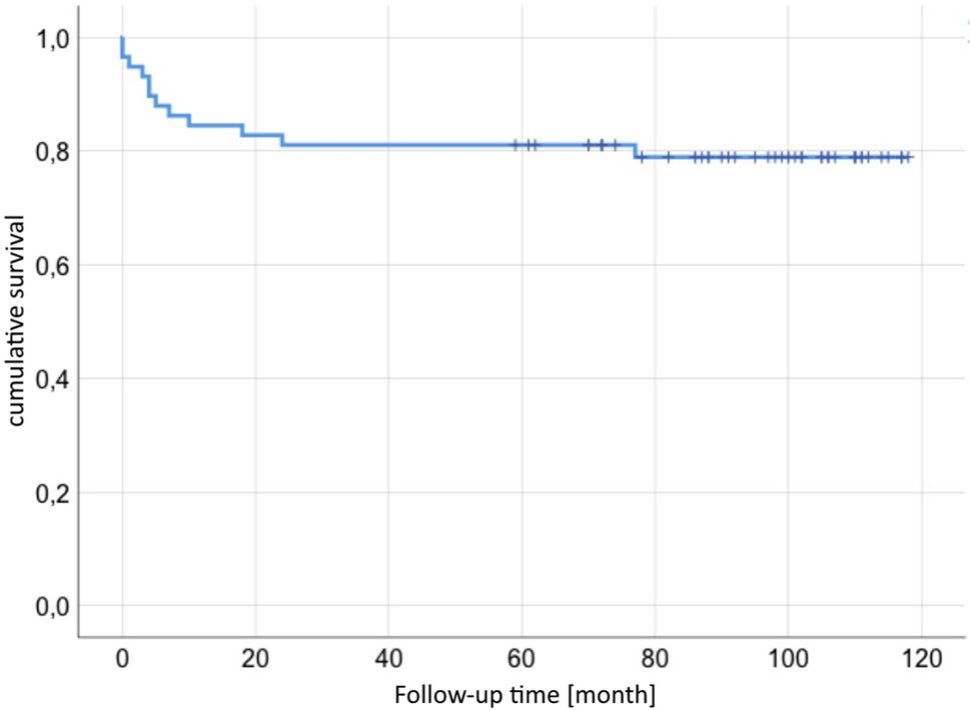
Kaplan–Meier-curve for general cup survival.

**Figure 6:**
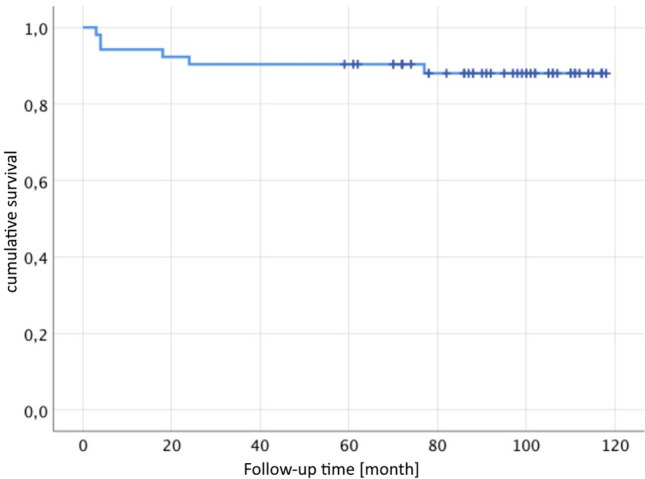
Kaplan–Meier-curve for cup survival based on aseptic loosening or acetabular fracture.

**Figure 7:**
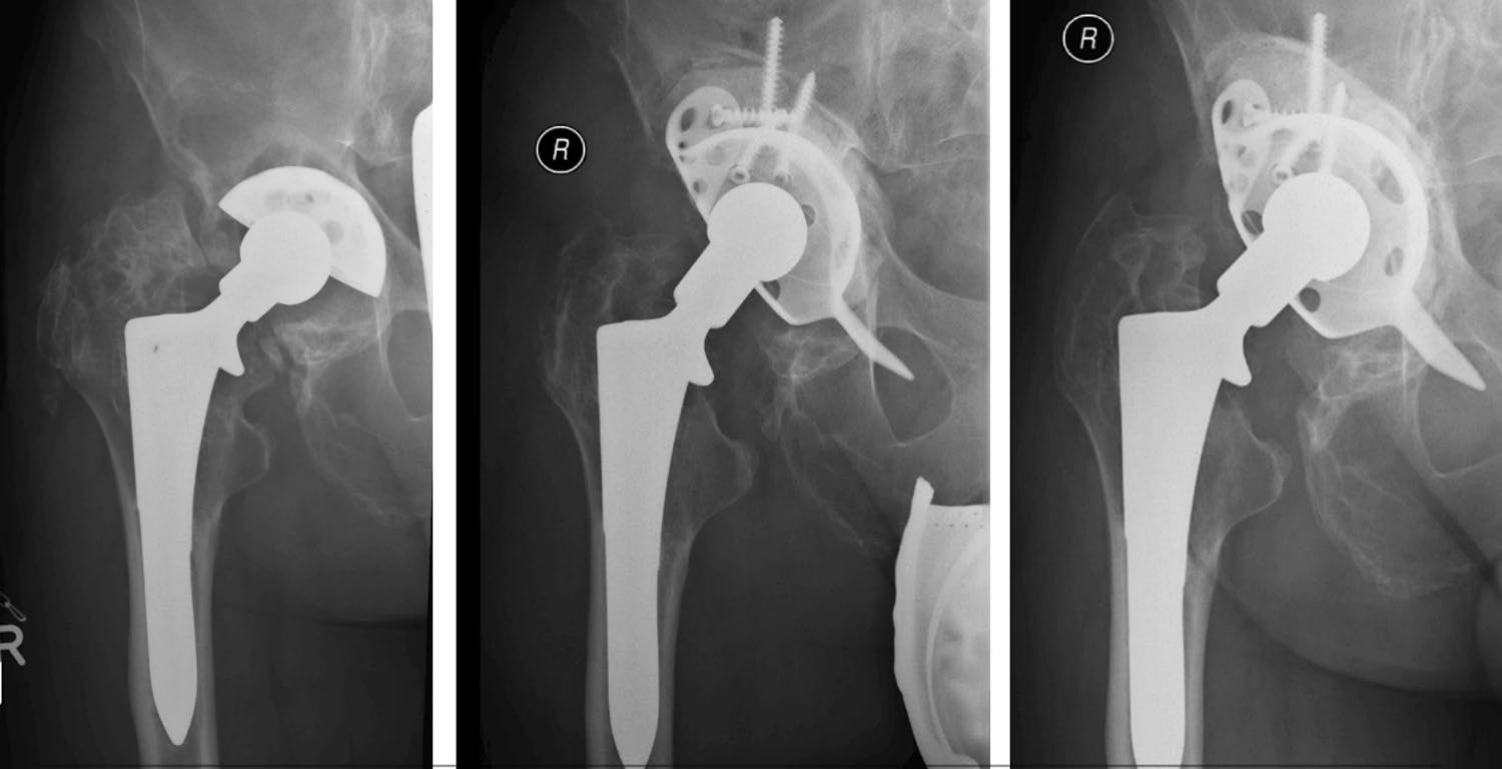
Preoperative Paprosky type 3a defect and postoperative controls after 1 week and 6 years.

**Figure 8:**
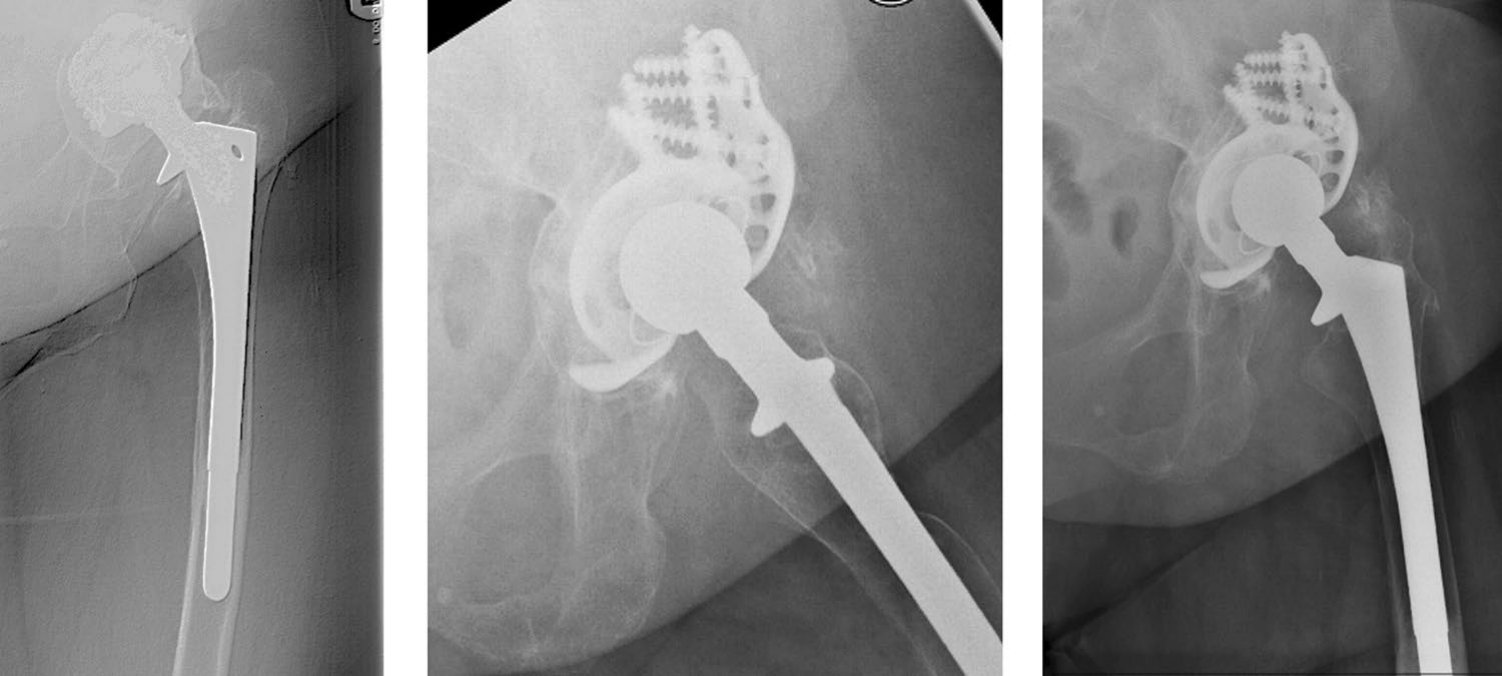
Preoperative Paprosky type 3a defect and postoperative controls after 1 week and 7.6 years.

1. Defining aseptic loosening and periprosthetic fracture as failure of the bone graft, there is a revision free survival after 5 years of 90% and after 7.8 years of 88% (Figs. 5, 6). Figures 7, 8 and 9 show two successfull and one unsuccessfull bone graft reconstruction of acetabular defects (Figs. 7, 8, 9).

**Figure 9:**
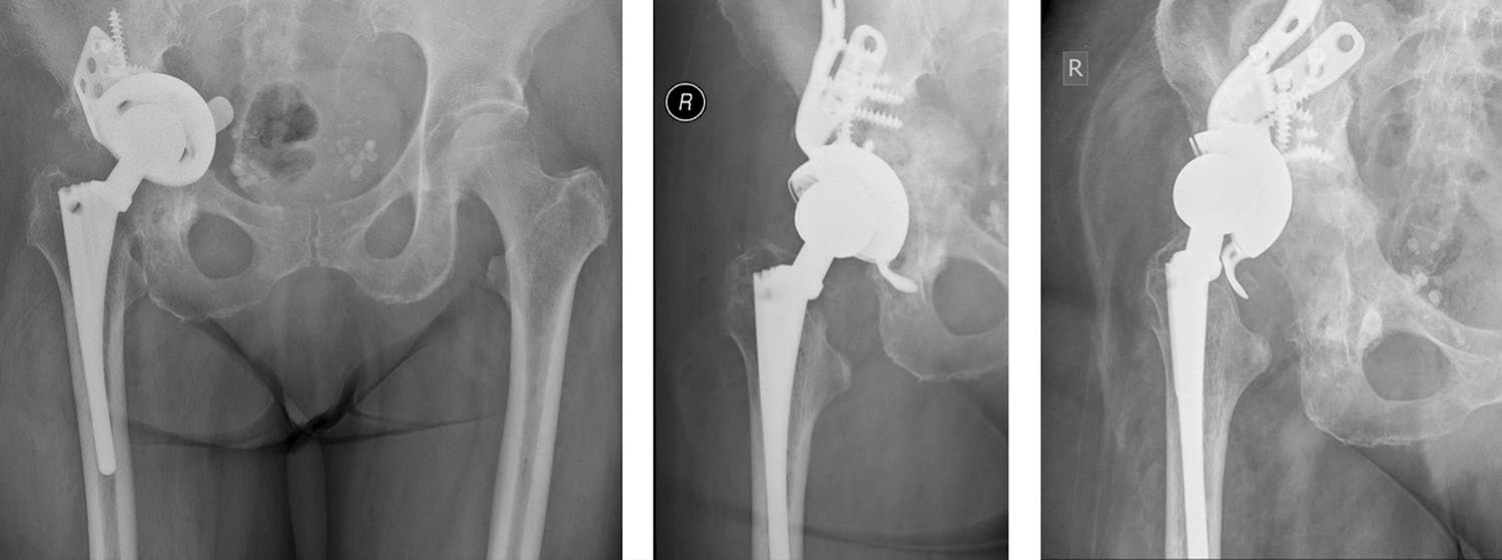
Preoperative Paprosky type 3a defect and postoperative controls after 1 week and 1.5 years with renewed cup loosening.
2. Furthermore, there was no significant difference in survival concerning defect classification (*p* = 0.853), type of implant (*p* = 0.882) or graft (*p* = 0.958), age (*p* = 0.456), BMI (*p* = 0.910), and number of previous operations (*p* = 0.517). As well spearman-rho correlation analysis showed no significant correlation between these factors and aseptic loosening. Periprosthetic infection (n = 4) occurred only in bulk grafts in our study group and 5 out of 6 aseptic loosening occurred within the first 2 years.

### Follow up data

(3) Patients on the follow up reached an HHS of 67.4 ± 19 points (of 100 points). Subdivided in pain and functional score our patients had 30.4 ± 11.4 (of 44) points regarding on pain and 29.8 ± 11.6 (of 47) points regarding on function.

On WOMAC Score they reached 33.4 ± 25.4 points. Subdivided into pain, stiffness and restrictions of everyday activity patients reached 22.5 ± 25.6 points for pain, 35.0 ± 28.4 points for stiffness and 36.4 ± 27.9 points for restrictions of everyday activity while less points mean a better outcome.

Table 2 shows the subdivided results of the SF-36 from our patients compared to a German normal and age-adjusted baseline population.

**Table 2:** SF-36 questionnaire.

	Study sample			German normal sample			German normal sample age > 70 years		
	N	Mean ± SD	z-value	N	Mean ± SD	*p*	N	Mean ± SD	*p*
KOEFU	16	46.88 ± 32.96	− 1.84	2886	85.71 ± 22.10	**< 0.0001**	326	58.59 ± 27.44	0.0998
KOERO	16	37.50 ± 43.78	− 1.25	2856	83.70 ± 31.73	**< 0.0001**	317	62.16 ± 40.80	**0.0193**
SCHM	16	48.44 ± 30.38	− 1.11	2905	79.08 ± 27.38	**< 0.0001**	327	64.20 ± 28.13	**0.0299**
AGES	16	43.06 ± 18.15	− 1.48	2859	68.05 ± 20.15	**< 0.0001**	324	55.30 ± 20.96	**0.0225**
VITA	16	49.06 ± 20.10	− 0.51	2876	63.27 ± 18.47	**0.0022**	324	53.91 ± 21.39	0.3753
SOFU	16	80.50 ± 30.62	− 0.18	2911	88.76 ± 18.40	0.0747	328	83.94 ± 21.27	0.5374
EMRO	16	64.50 ± 39.47	− 0.67	2855	90.35 ± 25.62	**< 0.0001**	320	83.04 ± 33.72	**0.034**
PSYC	16	65.00 ± 19.57	− 0.54	2871	73.88 ± 16.38	**0.0308**	324	71.41 ± 17.21	0.1494

Significant values are in [bold].

*KOEFU* physical function, *KOERO* physical role, *SCHM* bodily pain, *AGES* general health, *VITA* vitality, *SOFU* social function, *EMRO* emotional role, *PSYC* mental health.

## Discussion

Reconstruction of large acetabular defects in revision hip arthroplasties continues to constitute a great challenge. Surgical options include next to bone grafts, extra-large hemispheric cups^25^, reconstruction cages^26^ and more recent techniques like trabecular metal implants and augments^9^. Bone impaction grafting of the acetabulum had been advocated as an excellent method for reconstructing acetabular defects and for restoration acetabular bone stock and anatomical COR with good long-term survival^19,27^. But there is still a controversy which technique to use respective to the defect size.

(2) Our results showed no dependence between survival and defect classification as well as type of implant or graft, age, BMI, and number of previous operations. Furthermore, reconstruction with bulk grafts showed no increased failure than cortico-cancellous grafts. Even reconstruction cages showed no increased failure than cemented or cementless cups in our study. Also, Patel et al. reported no single factor influencing the cup survival, although type III defects showed a higher migration rate of the cup^28^. But there is still a lack of knowledge about influencing factors to implant survival due to small case numbers and individual surgical strategies.
(1) Our study confirmed that an allogenic bone reconstruction of the acetabulum can lead to satisfactory biomechanical results. We showed a survival in Kaplan-Meyer-analyses of 88% for aseptic loosening after 7.8 years with allogenic acetabular impaction grafting and subsequent implantation of cups and reconstruction cages without significant differences in-between the specific implants. Concerning reconstruction cages, we confirmed the results of Perka et al. who reported a survival rate of 88.8% after 10 years for Paprosky type IIa-IIIb defects treated with Burch-Schneider cages^29^. Concerning jumbo or extra-large cups Roth et al. also reported survival rates of 85% after 20 years^30^. An important disadvantage of jumbo cups is the elevation of the hip centre of rotation which can sometimes be handled with a head enlargement^31^. For bulk allografts a survival of 56% after 4 years^20^ and 39% after 16.5 years with cemented cups was reported^32^. For bulk allografts with uncemented cups Paprosky et al. reported a survival of 80% after 10 years for Paprosky type IIIa defects^21^. Bulk allografts with reconstruction cages showed a survival of 84 to 98%^33–36^ in follow up studies between 5 and 19 years, confirming our results with a survivor of 88% (*p* = 0.958).
  Next to extra-large cups and reconstruction cage, trabecular metal implants enjoy increasing popularity^37^. First studies showed an excellent survival rate > 90% for Paprosky type IIa–IIIb defects after 5 and 10 years^10,38,39^. The disadvantage of trabecular metal augments is a missing bony replenishment of the defect. In case of potential revision surgery, a larger defect can occur after explanting the metal augment. Furthermore, there is less known about the effect of the potential wearing of the trabecular metal augments. Elevated level of metal particles in the periprosthetic soft tissue and blood samples are reported after aseptic loosening and revision surgery^40–42^. But recent studies showed that especially for larger defects (Paprosky IIIa, b) trabecular metal augments show a good outcome and could be recommend^11,43^. Furthermore, Shen et al. showed in a meta-analysis no difference in survival of trabecular metal and non-trabecular metal cups in acetabular revision surgery overall, but with a lower incidence of aseptic loosening and infection and a higher incidence of dislocation for trabecular metal compared to non-trabecular metal cups^11^. Finally, a comparison between the studies remains difficult because of the use of different defect classification and implants, surgical experiences as well as small sample numbers (Table 3).

**Table 3:** Review of cup survival in literature.

	Follow up (y)	N	Classification	Cup survival/failure rate
**Uncemented cups and cortico-cancellous bone grafts**				
Della Valle et al.^14^	15	138	Pap. I–IIIB	Survival: 81%
				Aseptic Survival: 96%
Jones and Lachiewicz^15^	12	211	AAOS I–IV	Survival: 95%
				Aseptic Survival: 98%
**Reconstruction cages and cortico-cancellous bone grafts**				
Perka and Ludwig^29^	5.5 (3–10)	63	Pap. IIA–IIIB	Survival: 88.8%
				Aseptic loosening: 4.8%
Winter et al.^47^	7.3	38	AAOS II, IV	Aseptic loosening: 0%
Marx et al.^18^	7	74	AAOS III	Aseptic loosening: 6.2%
Wachtl et al.^48^	12 (8–21)	38	AAOS II–IV	Survival 92% (21 y)
**Reconstruction cages and solid bone grafts**				
Schlegel et al.^33^	6 (2–17)	122	AAOS I–IV	Aseptic survival: 98% (5 y), 95% (8 y)
Gerber et al.^34^	9 (7.8–11.6)	50	AAOS II–IV	Survival: 81% (10 y)
Regis et al.^35^	11.7 (10–14.4)	56	Pap. IIIA–IIIB	Survival: 87.5%
				Aseptic loosening: 8.9%
Regis et al.^36^	14.6 (10–18.9)	65	Pap. IIIA–IIIB	Survival: 80% (18.9 y)
				Aseptic survival: 84.6% (18.9 y)
**Trabecular metal revision systems**				
Whitehouse et al.^39^	7–11	40	Pap. IIA–IIIB	Survival: 87% (10 y)
				Survival graft: 92% (10 y)
Jenkins et al.^38^	5–12.5	58	Pap. IIA–IIIB	Survival: 100% (5 y), 97% (10 y)
Löchel et al.^10^	10	53	Pap. IIA–IIIB	Survival: 92.5%
(3) Our study population showed a poor functionality with an HHS of 67.4 points. Regis et al. and Wedemeyer et al. reported better functional outcomes (HHS > 75 points) in their studies with comparable defects after 7 and 10 years^35,44^. But their patients had a lower average age of 65 years in comparison to 70.3 years in our study. In our study there was no correlation between functional outcome measured in HHS and defect size (*p* = 0.745)^45^. Concerning the WOMAC- and SF-36-Score Sembrano et al. confirmed our results of 33 points in WOMAC and 65 points in psychological and 48 points in functional SF-36-score^46^.

Limitations of the study were the small number of cases with several types of cups or cages and a variability in patients’ condition without a control group in a retrospective study design and a mid-term follow up of only 7.8 years. Another limitation was the classification in Paprosky defect type through the pre-operative X-ray. Further intraoperative damage on the acetabulum was not verified. Furthermore, comparison remains difficult because of different defect classification and implants, small case numbers and the surgeons experience. As well the missing preoperative functional score and baseline characteristics of the joint function without drawing the course of the score and joint characteristics pre- and postoperatively is a limitation.

## Conclusion

In conclusion bone grafting of acetabular defects for reconstruction of the COR is a validated and a good option with satisfactory biomechanical results and a good survival, especially in smaller acetabular defects. There could be no predictive factors for cup survival clarified in our study group. Neither pre-existing operation, defect classification nor implant model showed a significant influence on survival or outcome. In the end the correct indication, knowing the limits of the methods and the correct choice of implant allow a defect-oriented approach and are decisive for the success of the operation.
